# Stroke Mortality Among Black and White Adults Aged ≥35 Years Before and During the COVID-19 Pandemic — United States, 2015–2021

**DOI:** 10.15585/mmwr.mm7216a4

**Published:** 2023-04-21

**Authors:** Quanhe Yang, Xin Tong, Linda Schieb, Fátima Coronado, Robert Merritt

**Affiliations:** 1 Division for Heart Disease and Stroke Prevention, National Center for Chronic Disease Prevention and Health Promotion, CDC.

Stroke is the fifth leading cause of death and a leading cause of long-term disability in the United States (*1*). Although stroke death rates have declined since the 1950s, age-adjusted rates remained higher among non-Hispanic Black or African American (Black) adults than among non-Hispanic White (White) adults (*1*,*2*). Despite intervention efforts to reduce racial disparities in stroke prevention and treatment through reducing stroke risk factors, increasing awareness of stroke symptoms, and improving access to treatment and care for stroke (*1*,*3*), Black adults were 45% more likely than were White adults to die from stroke in 2018.* In 2019, age-adjusted stroke death rates (AASDRs) (stroke deaths per 100,000 population) were 101.6 among Black adults and 69.1 among White adults aged ≥35 years. Stroke deaths increased during the early phase of the COVID-19 pandemic (March–August 2020), and minority populations experienced a disproportionate increase (*4*). The current study examined disparities in stroke mortality between Black and White adults before and during the COVID-19 pandemic. Analysts used National Vital Statistics System (NVSS) mortality data accessed via CDC WONDER^†^ to calculate AASDRs among Black and White adults aged ≥35 years prepandemic (2015–2019) and during the pandemic (2020–2021). Compared with that during the prepandemic period, the absolute difference in AASDR between Black and White adults during the pandemic was 21.7% higher (31.3 per 100,000 versus 38.0). During the pandemic period, an estimated 3,835 excess stroke deaths occurred among Black adults (9.4% more than expected) and 15,125 among White adults (6.9% more than expected). These findings underscore the importance of identifying the major factors contributing to the widened disparities; implementing prevention efforts, including the management and control of hypertension, high blood cholesterol, and diabetes; and developing tailored interventions to reduce disparities and advance health equity in stroke mortality between Black and White adults. Stroke is a serious medical condition that requires emergency care. Warning signs of a stroke include sudden face drooping, arm weakness, and speech difficulty. Immediate notification of Emergency Medical Services by calling 9-1-1 is critical upon recognition of stroke signs and symptoms.

Annual AASDRs (calculated using 2000 U.S. Census Bureau standard population) and 95% CIs for Black and White adults aged ≥35 years were calculated for 2015–2020 (using final underlying cause of death data stratified by bridged-race categories) and 2021 (using underlying cause of death data stratified by single-race categories as of March 20, 2023) using CDC WONDER mortality data based on place of residence data from death certificates filed in the 50 U.S. states and the District of Columbia. AASDRs and 95% CIs were then calculated for the prepandemic and pandemic periods. *International Classification of Diseases, Tenth Revision* cause of death codes I60–I69 (cerebrovascular disease) were used to classify stroke as the underlying cause of death. This study included persons listed as having one race (Black or White) and non-Hispanic or Latino ethnicity.^§^ Disparities between Black and White adults were measured using absolute and relative differences. The absolute difference in AASDR among adults aged ≥35 years (rate among Black adults minus rate among White adults) was calculated for the prepandemic and pandemic periods and compared. The relative difference was measured by rate ratios calculated as AASDR among Black adults divided by AASDR among White adults. The 95% CIs for the absolute and relative differences were estimated. Nonoverlapping 95% CIs for rates, absolute differences, or relative differences between two periods or two groups (e.g., women versus men) were considered statistically significant. Analyses were stratified by sex and age group (35–64, 65–84, and ≥85 years).

Excess stroke deaths for Black and White adults during the pandemic were estimated as follows: 1) annual percent changes (APC) in the sex-, age-, and race and ethnicity–specific stroke death rates during 2015–2019 were estimated; 2) expected rates for 2020 and 2021 were calculated, assuming the APC during 2015–2019 would continue during 2020–2021; 3) the expected number of stroke deaths was obtained by multiplying the sex-, age-, and race and ethnicity–specific population by the expected stroke death rates; and 4) excess stroke deaths were calculated as the number of stroke deaths observed minus the number expected^¶^ (*5*). Analyses were conducted using SAS software (version 9.4; SAS Institute) and Joinpoint (version 4.8.1.0; National Cancer Institute). This activity was reviewed by CDC and was conducted consistent with applicable federal law and CDC policy.**

During 2015–2019, AASDR remained consistent for both Black (range = 100.5–102.0 [with a slight trend of increased rates]) and White adults (range = 69.1–70.6 [with a slight trend of decreased rates]); the average absolute difference was 31.3 per 100,000, and the average relative difference was 1.4. The relative differences in stroke mortality between Black and White adults were higher among men than women, and decreased with increasing age (Table 1) (Figure). During the COVID-19 pandemic, AASDR increased among both populations, but the increase was larger among Black adults; the average AASDR among Black adults increased 11.2% (from 101.3 to 112.6), compared with 6.6% (from 70.0 to 74.6) among White adults. The absolute difference in AASDR between Black and White adults increased 21.7% (from 31.3 to 38.0), and the average relative difference increased to 1.5. The patterns of increased disparities in stroke mortality, as measured by percent change in absolute differences, during the pandemic were similar among men and women and increased with increasing age. The absolute difference in AASDR between Black and White adults increased 10.9% (from 16.6 to 18.4) among persons aged 35–64 years and 19.4% (from 89.8 to 107.2) among persons aged 65–84 years. During the prepandemic period, annual stroke mortality rates among persons aged ≥85 years were lower among Black than among White adults (*2*). The absolute difference in this age group changed from −23.7 during 2015–2019 to 30.3 per 100,000 during 2020–2021 (Table 1). During 2020–2021, an estimated 3,835 excess stroke deaths occurred among Black adults (9.4% more than expected), and 15,125 excess stroke deaths occurred among White adults (6.9% more than expected). The estimated percentage of excess stroke deaths among both Black and White adults was higher among women and decreased with increasing age (Table 2).

**TABLE 1:** Age-adjusted stroke death rates among Black and White adults* aged ≥35 years before (2015–2019) and during (2020–2021) the COVID-19 pandemic, by age group and sex — United States, 2015–2021

Characteristic	Age-adjusted stroke death rates per 100,000^†^ (95% CI)^§^									% Change in absolute differences between Black and White adults
	Pre–COVID-19						During COVID-19			
	2015	2016	2017	2018	2019	Average 2015–2019	2020	2021	Average 2020–2021	2020–2021 vs. 2015–2019^¶^
**Total**										
**BNH**	**100.9 (99.4 to 102.5)**	**100.5 (99.0 to 102.0)**	**102.0 (100.5 to 103.5)**	**101.3 (99.8 to 102.7)**	**101.6 (100.1 to 103.0)**	**101.3 (100.6 to 101.9)**	**110.0 (108.5 to 111.4)**	**115.4 (113.9 to 117.0)**	**112.6 (111.6 to 113.7)**	**—**
**WNH**	**70.6 (70.2 to 71.1)**	**70.1 (69.7 to 70.5)**	**70.6 (70.2 to 71.1)**	**69.7 (69.3 to 70.1)**	**69.1 (68.7 to 69.5)**	**70.0 (69.8 to 70.2)**	**72.0 (71.6 to 72.5)**	**77.4 (76.9 to 77.8)**	**74.6 (74.3 to 74.9)**	**—**
**Absolute difference, BNH vs. WNH**	**30.3 (28.7 to 31.9)**	**30.4 (28.9 to 32.0)**	**31.3 (29.8 to 32.9)**	**31.6 (30.1 to 33.1)**	**32.4 (30.9 to 33.9)**	**31.3 (31.0 to 31.5)**	**37.9 (36.3 to 39.5)**	**38.0 (36.4 to 39.7)**	**38.0 (37.0 to 39.1)**	**21.7 (18.0 to 25.3)**
Relative difference, BNH vs. WNH**	**1.4 (1.4 to 1.5)**	**1.4 (1.4 to 1.5)**	**1.4 (1.4 to 1.5)**	**1.5 (1.4 to 1.5)**	**1.5 (1.4 to 1.5)**	**1.4 (1.4 to 1.5)**	**1.5 (1.5 to 1.5)**	**1.5 (1.5 to 1.5)**	**1.5 (1.5 to 1.5)**	**—**
**Sex**										
**Men**										
BNH	110.3 (107.7 to 112.9)	109.8 (107.2 to 112.4)	112.1 (109.5 to 114.6)	112.7 (110.2 to 115.2)	110.8 (108.3 to 113.2)	**111.2 (110.0 to 112.3)**	122.6 (120.0 to 125.1)	125.7 (123.1 to 128.3)	**124.1 (122.3 to 125.9)**	—
WNH	69.6 (68.9 to 70.3)	69.2 (68.5 to 69.8)	69.9 (69.2 to 70.5)	69.1 (68.5 to 69.8)	69.0 (68.4 to 69.6)	**69.3 (69.0 to 69.6)**	72.4 (71.7 to 73.0)	76.7 (76.0 to 77.4)	**74.4 (74.0 to 74.9)**	—
Absolute difference, BNH vs. WNH	40.7 (38.0 to 43.4)	40.6 (38.0 to 43.3)	42.2 (39.5 to 44.8)	43.6 (41.0 to 46.2)	41.8 (39.2 to 44.3)	**41.8 (40.7 to 43.0)**	50.2 (47.6 to 52.8)	49.0 (46.3 to 51.6)	**49.7 (47.9 to 51.5)**	18.7 (13.2 to 24.4)
Relative difference, BNH vs. WNH**	1.6 (1.5 to 1.6)	1.6 (1.5 to 1.6)	1.6 (1.6 to 1.6)	1.6 (1.6 to 1.7)	1.6 (1.6 to 1.6)	**1.6 (1.6 to 1.6)**	1.7 (1.7 to 1.7)	1.6 (1.6 to 1.7)	**1.7 (1.6 to 1.7)**	—
**Women**										
BNH	92.7 (90.8 to 94.5)	92.6 (90.7 to 94.4)	93.5 (91.7 to 95.4)	91.9 (90.2 to 93.7)	93.5 (91.7 to 95.3)	**92.9 (92.0 to 93.7)**	99.5 (97.7 to 101.3)	106.3 (104.4 to 108.2)	**102.8 (101.5 to 104.2)**	—
WNH	70.3 (69.7 to 70.9)	69.5 (68.9 to 70.1)	70.0 (69.5 to 70.6)	69.0 (68.5 to 69.5)	68.1 (67.6 to 68.6)	**69.4 (69.1 to 69.6)**	70.6 (70.1 to 71.2)	76.7 (76.1 to 77.3)	**73.5 (73.1 to 73.9)**	—
Absolute difference, BNH vs. WNH	22.4 (20.4 to 24.3)	23.1 (21.2 to 25.0)	23.5 (21.6 to 25.4)	22.9 (21.1 to 24.8)	25.4 (23.5 to 27.2)	**23.5 (22.6 to 24.3)**	28.9 (27.0 to 30.8)	29.6 (27.6 to 31.6)	**29.3 (27.9 to 30.7)**	24.8 (17.5 to 32.3)
Relative difference, BNH vs. WNH**	1.3 (1.3 to 1.3)	1.3 (1.3 to 1.4)	1.3 (1.3 to 1.4)	1.3 (1.3 to 1.4)	1.4 (1.3 to 1.4)	**1.3 (1.3 to 1.4)**	1.4 (1.4 to 1.4)	1.4 (1.4 to 1.4)	**1.4 (1.4 to 1.4)**	—
**Age group, yrs**										
**35–64**										
BNH	28.0 (27.2 to 28.8)	27.4 (26.6 to 28.2)	27.6 (26.8 to 28.3)	27.0 (26.3 to 27.8)	27.0 (26.3 to 27.8)	**27.4 (27.1 to 27.8)**	30.4 (29.5 to 31.2)	30.9 (30.1 to 31.7)	**30.6 (30.0 to 31.2)**	—
WNH	10.6 (10.4 to 10.8)	10.8 (10.6 to 11.1)	10.8 (10.6 to 11.0)	10.8 (10.6 to 11.0)	10.9 (10.7 to 11.2)	**10.8 (10.7 to 10.9)**	12.0 (11.7 to 12.2)	12.4 (12.2 to 12.7)	**12.2 (12.0 to 12.4)**	—
Absolute difference, BNH vs. WNH	17.4 (16.6 to 18.2)	16.6 (15.8 to 17.4)	16.8 (16.0 to 17.6)	16.2 (15.4 to 17.0)	16.1 (15.3 to 16.9)	**16.6 (16.3 to 17.0)**	18.4 (17.8 to 19.0)	18.5 (17.6 to 19.3)	**18.4 (18.0 to 18.8)**	10.9 (6.6 to 15.4)
Relative difference, BNH vs. WNH**	2.6 (2.5 to 2.7)	2.5 (2.4 to 2.6)	2.6 (2.5 to 2.6)	2.5 (2.4 to 2.6)	2.5 (2.4 to 2.6)	**2.5 (2.5 to 2.6)**	2.5 (2.5 to 2.6)	2.5 (2.4 to 2.6)	**2.5 (2.5 to 2.6)**	—
**65–84**										
BNH	236.1 (230.9 to 241.3)	232.1 (227.0 to 237.2)	235.1 (230.1 to 240.1)	233.6 (228.7 to 238.5)	233.7 (228.9 to 238.4)	**234.1 (231.9 to 236.3)**	249.0 (244.2 to 253.8)	264.3 (259.2 to 269.3)	**256.6 (253.1 to 260.1)**	—
WNH	148.4 (147.0 to 149.8)	146.0 (144.6 to 147.4)	145.2 (143.8 to 146.5)	141.8 (140.4 to 143.1)	140.8 (139.5 to 142.1)	**144.3 (143.7 to 144.9)**	145.8 (144.5 to 147.1)	153.1 (151.7 to 154.4)	**149.4 (148.5 to 150.3)**	—
Absolute difference, BNH vs. WNH	87.7 (82.2 to 93.1)	86.1 (80.9 to 91.3)	90.0 (84.8 to 95.2)	91.8 (86.7 to 96.9)	92.9 (87.9 to 97.9)	**89.8 (87.5 to 92.1)**	103.2 (98.2 to 108.2)	111.2 (105.9 to 116.4)	**107.2 (103.5 to 110.7)**	19.4 (14.5 to 24.5)
Relative difference, BNH vs. WNH**	1.6 (1.6 to 1.6)	1.6 (1.6 to 1.6)	1.6 (1.6 to 1.7)	1.6 (1.6 to 1.7)	1.7 (1.6 to 1.7)	**1.6 (1.6 to 1.6)**	1.7 (1.7 to 1.7)	1.7 (1.7 to 1.8)	**1.7 (1.7 to 1.7)**	—
**≥85**										
BNH	944.3 (916.3 to 972.2)	973.4 (945.4 to 1,001.4)	995.6 (967.8 to 1,023.5)	997.2 (969.8 to 1,024.7	1,005.7 (978.5 to 1,033.0)	**984.1 (971.7 to 996.5)**	1,090.3 (1,062.3 to 1,118.4)	1,148.0 (1,117.7 to 1,178.2)	**1,117.7 (1,097.2 to 1,138.3)**	—
WNH	1,003.8 (995.1 to 1,012.4)	997.5 (988.9 to 1,006.1)	1,022.8 (1,014.1 to 1,031.4)	1,014.9 (1,006.2 to 1,023.5)	1,000.0 (991.4 to 1,008.6)	**1,007.8 (1,003.9 to 1,011.7)**	1,035.0 (1,026.3 to 1,043.8)	1,147.0 (1,137.2 to 1,156.7)	**1,087.5 (1,080.9 to 1,094.0)**	—
Absolute difference, BNH vs. WNH	−59.5 (−89.0 to 30.5)	−24.1 (−53.4 to 5.2)	−27.1 (−56.3 to 2.3)	−17.7 (−46.4 to 11.4)	5.7 (−22.8 to 34.4)	**−23.7 (−36.7 to 10.5)**	55.3 (25.9 to 84.2)	1.0 (30.6 to 32.4)	**30.3 (8.3 to 51.4)**	227.5 (133.8 to 426.9)
Relative difference, BNH vs. WNH**	0.9 (0.9 to 1.0)	1.0 (0.9 to 1.0)	1.0 (0.9 to 1.0)	1.0 (1.0 to 1.0)	1.0 (1.0 to 1.0)	**1.0 (1.0 to 1.0)**	1.1 (1.0 to 1.1)	1.0 (1.0 to 1.0)	**1.0 (1.0 to 1.1)**	—

**Abbreviations**: AASDR = age-adjusted stroke death rate; BNH = Black or African American, non-Hispanic; WNH = White, non-Hispanic.

* Persons listed as BNH or WNH (not including those with race listed as “more than one race” or Hispanic ethnicity listed as “not stated”) were included in this study.

^†^ Per 100,000 persons, standardized to the 2000 U.S. Census Bureau population by age group (35–54, 55–64, 65–74, 75–84, and ≥85 years).

^§^ For AASDRs, 95% CIs are calculated using Anderson-Rosenberg methods with the normal approximations of the 95% CIs (https://www.cdc.gov/nchs/data/nvsr/nvsr47/nvs47_03.pdf). For absolute and relative differences in rates, and percent change of absolute difference in rates between BNH and WNH adults (2020–2021 versus 2015–2019), 95% CIs are estimated by the Monte Carlo simulation–based approach by sampling 5,000 normal distributions based on the AASDRs and SEs and defining the CIs as the 2.5 and 97.5 percentiles of the simulation results using @Risk software (version 8.3.2; Palisade). https://help.palisade.com/v8/en/Guides/@RISK-Getting-Started-Guide.pdf

^¶^ Percent change of absolute difference in rates in 2015–2019 versus 2020–2021 between BNH and WNH adults is calculated by 1) calculating the difference of the average absolute difference in rates between BNH and WNH adults in 2020–2021 and the average absolute difference in rates between BNH and WNH adults in 2015–2019, and 2) dividing the above difference by the average absolute difference in rates between BNH and WNH adults in 2015–2019 and multiplying by 100.

** Relative difference in rates are AASDRs among BNH divided by AASDRs among WNH adults. The percent change in absolute differences between BNH and WNH adults in 2015–2019 compared with 2020–2021 might be different based on the results presented in the table because of rounding the results to one-decimal point.

**FIGURE:**
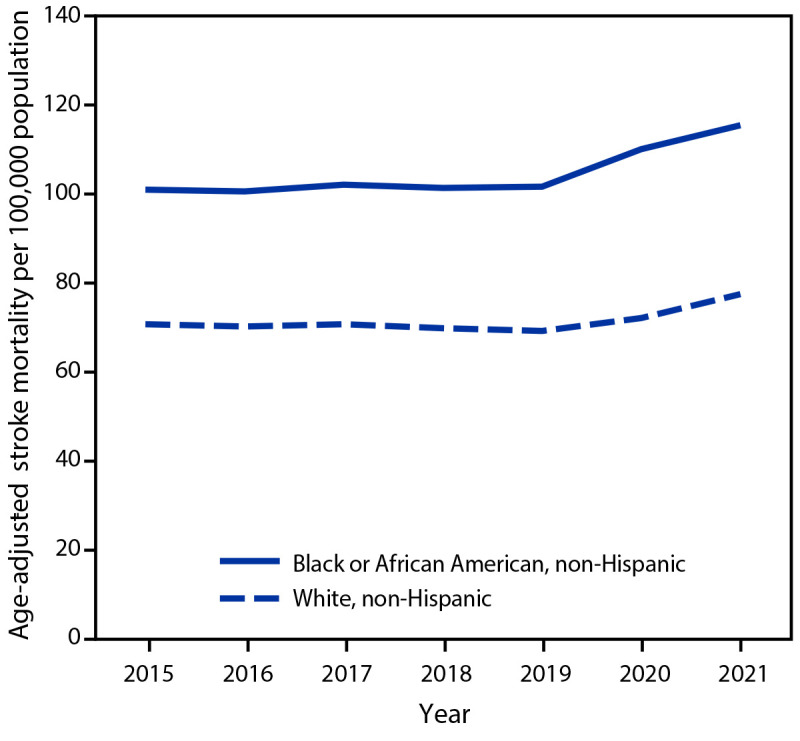
Age-adjusted stroke death rates* per 100,000 Black and White adults^†^ aged ≥35 years, before and during^§^ the COVID-19 pandemic — United States, 2015–2021 * Per 100,000 persons, standardized to the 2000 U.S. Census Bureau population by age group (35–54, 55–64, 65–74, 75–84, and ≥85 years). ^†^ Persons listed as Black or African American or White (not including those listed as “more than one race”) and listed as non-Hispanic or Latino (not including those with Hispanic ethnicity listed as “not stated”) were included in this study. ^§^ The period before the pandemic was defined as 2015–2019 and the period during the pandemic as 2020–2021.

**TABLE 2:** Observed, expected, and estimated number and percentage of excess stroke deaths among Black and White adults* aged ≥35 years during the COVID-19 pandemic, by age group and sex — United States, 2020–2021

Characteristic	Stroke deaths, 2020–2021					
	Black or African American, non-Hispanic			White, non-Hispanic		
	Observed, no.	Expected,^†^ no.	Excess,^§^ no. (%)	Observed, no.	Expected,^†^ no.	Excess,^§^ no. (%)
**Total**	**44,686**	**40,851**	**3,835 (9.4)**	**233,639**	**218,514**	**15,125 (6.9)**
**Sex**						
Men	20,407	18,705	1,702 (9.1)	99,207	93,653	5,554 (5.9)
Women	24,279	22,146	2,133 (9.6)	134,432	124,861	9,571 (7.7)
**Age group, yrs**						
35–64	11,279	10,181	1,098 (10.8)	24,463	22,775	1,688 (7.4)
65–84	22,063	20,161	1,902 (9.4)	102,120	94,848	7,272 (7.7)
≥85	11,344	10,508	836 (8.0)	107,056	100,891	6,165 (6.1)

**Abbreviation**: AASDR = age-adjusted stroke death rate.

* Persons listed as Black or African American or White (not including those listed as “more than one race”) and non-Hispanic or Latino (not including those with Hispanic ethnicity listed as “not stated”) were included in this study.

^†^ The expected number of stroke deaths were obtained by 1) assuming that the sex-, age-, and race and ethnicity–specific stroke death rates would continue through 2021 at the annual rate of 2015–2019 as identified by joinpoint analysis and 2) multiplying the sex-, age-, and race and ethnicity–specific population with the expected sex-, age-, and race and ethnicity–specific stroke death rates for 2020 and 2021. The expected number of stroke deaths by sex and age group might not sum to the total number of expected stroke deaths because of rounding.

^§^ Excess stroke deaths were calculated based on the difference between the observed versus expected stroke deaths by sex, age, and race and ethnicity during 2020–2021. The percentages of excess stroke deaths were calculated by number of excess stroke deaths divided by number of expected stroke deaths multiplied by 100. The estimated excess number of stroke deaths by sex and age group might not sum to the total number of estimated excess stroke deaths because of rounding. The percentages of estimated excess stroke deaths among non-Hispanic Black or African American persons (9.4%) were lower than the changes in AASDR from prepandemic to during the pandemic periods (11.2%) because of the slight trend of increased AASDR during 2015–2019. The percentages of estimated excess stroke deaths among non-Hispanic White persons (6.9%) were slightly higher than the changes in AASDR from prepandemic to during the pandemic periods (6.6%) because of the slight trend of decreased AASDR during 2015–2019.

## Discussion

This analysis found that in the United States, disparities among Black and White adults in stroke mortality widened from the prepandemic period to the pandemic period. Although stroke mortality increased among both Black and White adults, the absolute difference in AASDRs between the groups increased an estimated 21.7%; this pattern was similar in men and women. The estimated percentage of excess stroke deaths during the pandemic period among Black adults (9.4%) was higher than that among White adults (6.9%). The disparity, measured by absolute difference in AASDR, among adults aged 35–54 years increased approximately 11%, and among adults aged 65–84 years, increased 19%; the lower stroke mortality among Black adults aged ≥85 years during the prepandemic period reversed during the pandemic period.

Disparities in stroke mortality among Black and White adults are largely driven by differences in stroke incidence, with higher prevalences of high blood pressure and diabetes being the major risk factors for stroke among Black adults (*1*,*2*,*6*); racial differences in case-fatality played a minor role (*2*,*6*). The COVID-19 pandemic caused a substantial shift in health care for patients with high blood pressure and might have exacerbated existing inequities in high blood pressure treatment and control among persons of color (*7*). Reduced emergency department visits and hospitalizations for stroke, partly because of fear of SARS-CoV-2 infections (especially during the early phase of the pandemic) (*8*), suggest that delayed stroke treatment and care might have resulted in worse stroke outcomes and increased risk for death. Further, health and lifestyle behaviors, such as mental health, physical activity, and diet and sleep quality were adversely affected by the pandemic and might have disproportionately affected persons of color, resulting in increased risk for stroke (*4*). COVID-19 is associated with increased risk for stroke (*9*); disproportionately higher rates of COVID-19 experienced by Black persons^††^ (*10*) might have contributed to the widened disparity among Black and White adults in stroke mortality. 

A main goal of the Healthy People 2030 initiative is to improve the health and well-being of all U.S. persons by eliminating health disparities, achieving health equity, and increasing health literacy.^§§^ Further studies are needed to identify and evaluate the underlying risk factors, including stress-related factors such as economic strain, poor mental health, and social determinants of health that might have contributed to the widened disparity between Black and White adults in stroke mortality during the COVID-19 pandemic. Tailored interventions to improve the prevention, control and management of risk factors, system-based stroke care, and structural changes addressing racial disparities in health care might be required to effect lasting change.

The findings in this report are subject to at least two limitations. First, the NVSS mortality data lacks information to determine how much of an increase in stroke mortality was directly attributable to the COVID-19 pandemic. Second, this study focused on disparity in stroke mortality among Black and White adults before and during the pandemic, and did not include other races.

Substantial disparities in stroke mortality between Black and White adults in the United States exist and have widened during the COVID-19 pandemic. The COVID-19 pandemic imposed setbacks to progress made in reducing disparities in stroke mortality between Black and White adults. Identifying factors associated with these widened disparities, implementing prevention efforts, including the management and control of stroke risk factors, preventing disparities in treatment and services for long term sequelae of stroke, and tailoring interventions to advance health equity are needed to reduce disparities in stroke mortality.

**Summary**

**What is already known about this topic?**

Stroke is the fifth leading cause of death and a leading cause of long-term disability in the United States. During 1999–2019, non-Hispanic Black or African American (Black) adults experienced consistently higher stroke death rates than did non-Hispanic White (White) adults.

**What is added by this report?**

During the COVID-19 pandemic, age-adjusted stroke mortality rates increased among both Black and White adults; however, the absolute difference between Black and White adults was 21.7% higher than during the prepandemic period. The percentage of excess stroke deaths during the pandemic was higher among Black (9.4%) than among White (6.9%) adults.

**What are the implications for public health practice?**

Identifying the health care, behavioral, and contextual factors associated with these widened disparities and providing tailored interventions are necessary to reduce disparities in stroke mortality among Black and White adults.

## Footnotes

*
https://www.heart.org/en/about-us/2024-health-equity-impact-goal/age-adjusted-total-stroke-mortality-rates-by-raceethnicity

† Data were accessed from the National Center for Health Statistics’ mortality data from 2018–2021 on the CDC WONDER online database, released in 2023. This study also selected 2015–2020 data from “1999–2020 Underlying Cause of Death by Bridged-race Categories” and 2021 data from “2018–2021 Underlying Cause of Death by Single-race Categories” and combined 2015–2020 and 2021 data for analyses. Mortailty data were compiled from data provided by the 57 vital statistics jurisdictions through the Vital Statistics Cooperative Program. https://wonder.cdc.gov/Deaths-by-Underlying-Cause.html

§ Persons listed as Black or White (not including those listed as “more than one race”) and listed as non-Hispanic or Latino (not including those with Hispanic ethnicity listed as “not stated”) were included in this study.

¶ Using different years of baseline data or different methods to predict 2020–2021 stroke death rates can result in different expected stroke death rates, and, therefore, the different estimated excess number of stroke deaths. https://www.cdc.gov/nchs/nvss/vsrr/covid19/excess_deaths.htm

** 45 C.F.R. part 46.102(l)(2), 21 C.F.R. part 56; 42 U.S.C. Sect. 241(d); 5 U.S.C. Sect. 552a; 44 U.S.C. Sect. 3501 et seq.

††
https://www.cdc.gov/coronavirus/2019-ncov/covid-data/investigations-discovery/hospitalization-death-by-race-ethnicity.html

§§
https://health.gov/healthypeople/priority-areas/health-equity-healthy-people-2030
